# Editorial: Immunological response to nanomaterials

**DOI:** 10.3389/fbioe.2024.1392097

**Published:** 2024-03-12

**Authors:** Luis Jesús Villarreal-Gómez, Giuseppe Bardi, José Manuel Cornejo-Bravo, Ma Concepción Peña-Juárez

**Affiliations:** ^1^ Facultad de Ciencias de la Ingeniería y Tecnología, Universidad Autónoma de Baja California Tijuana, Tijuana, Mexico; ^2^ Nanobiointeractions and Nanodiagnostics, Istituto Italiano di Tecnologia, Genova, Italy; ^3^ Facultad de Ciencias Químicas e Ingeniería, Universidad Autónoma de Baja California, Tijuana, Mexico; ^4^ Facultad de Estudios Superiores Cuautitlán, Universidad Nacional Autónoma de México, Cuautitlán Izcalli, Mexico

**Keywords:** immunological response, nanomaterials, nanocarriers, MicroRNAs, nanofibers, nanohydroxyapatite

Nanomaterials have emerged as promising tools in various fields, including medicine, electronics, and environmental remediation, owing to their unique physicochemical properties and versatile applications. However, as the utilization of nanomaterials continues to expand, concerns regarding their potential impact on human health and the environment have garnered increased attention, particularly in relation to the immunological response they may elicit (Asghar et al., 2024).

Understanding the interaction between nanomaterials and the immune system is essential for ensuring their safe and effective use. The immune system plays a crucial role in defending the body against foreign invaders and maintaining homeostasis. Therefore, any disruption or dysregulation of immune function by nanomaterials could have significant implications for human health (Ernst et al., 2021).

Research in the field of immunotoxicology has made significant strides in elucidating the effects of nanomaterials. Studies have shown that nanomaterial features, including size, shape, surface chemistry, and composition, can influence the immune response at different levels (Villarreal et al.) (Figure 1). For instance, certain nanoparticles have been found to trigger inflammatory responses, oxidative stress, and immunosuppression, while others may modulate immune cell function or elicit allergic reactions (Hofer et al., 2022).

**FIGURE 1:**
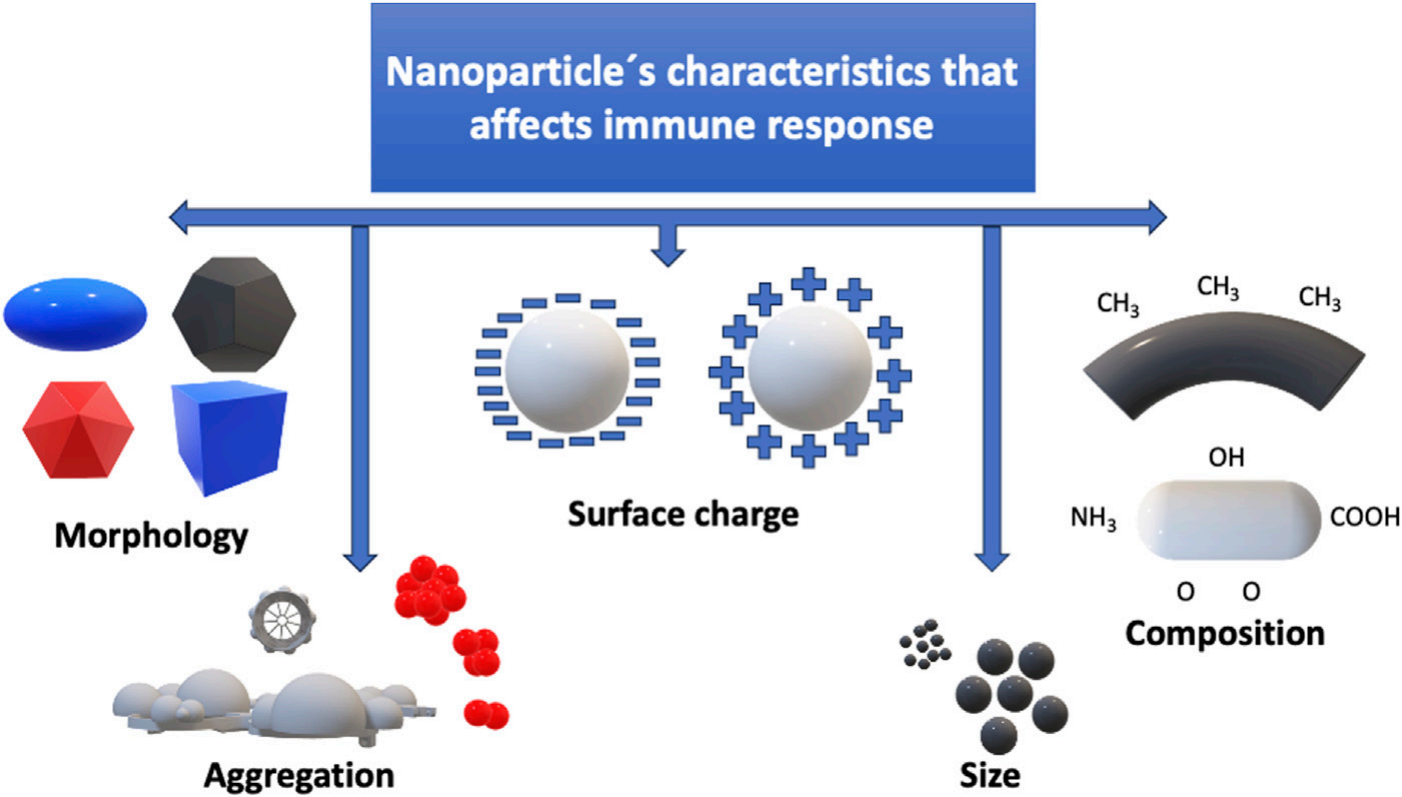
Attributes of nanoparticles that influence the body’s immune reactions.

Moreover, the route of exposure to nanomaterials can impact the nature and magnitude of the immune response. Inhalation, ingestion, dermal contact, and injection represent different routes through which nanomaterials can enter the body, each posing unique challenges and considerations for assessing immunotoxicity (Gupta and Xie, 2018).

This Research Topic addressed both the advantageous and detrimental immunological aspects associated with the presence of nanomaterials in various synthesized forms, including nanoparticles, nanogels, nanofibers, nanotubes, and others. These materials may serve as nanocarriers for drug delivery or substrates for tissue engineering and implantable biosensors.

Abdullah et al. studied a new carrier system for delivering anticancer drugs, comprising heat-inactivated *Lactiplantibacillus plantarum* (HILP) labeled with carbon dots (CDs), termed CDs/HILP. This hybrid carrier exhibited multifunctionality, acting as a probiotic drug carrier with bioimaging capabilities and utilizing prodigiosin (PG) as the anticancer agent. Preparation and characterization of HILP, CDs, and PG were conducted using established methods, including transmission electron microscopy (TEM) and laser scanning confocal microscopy (LSCM). The CDs/HILP system demonstrated sustained release of PG over 672 h and enhanced cytotoxicity against Caco-2 and A549 cells compared to free PG. Additionally, CDs/HILP facilitated improved distribution and nuclear delivery of PG, promoting late apoptosis of Caco-2 cells and reducing their migratory ability. Molecular docking studies suggested interaction between PG and mitogenic molecules involved in cell proliferation. Overall, CDs/HILP shows promise as an innovative, multifunctional nanobiotechnological carrier for delivering anticancer drugs (Abdullah et al.).

Vázquez et al. discussed that nanotechnologies offer significant potential for advancing miRNA-based cancer therapeutics, addressing current challenges and future opportunities. MicroRNAs (miRNAs), small non-coding RNA molecules, play a crucial role in cancer development by regulating gene expression post-transcriptionally. Despite efforts, targeted delivery of artificial miRNAs, such as anti-miRNAs and miRNA mimics, remains challenging. Nanotechnology-based delivery systems show promise in effectively delivering artificial miRNAs to target sites, offering innovative approaches to combat cancer initiation and progression. This review evaluates recent developments in nanotechnology-enabled miRNA delivery systems for cancer therapy and discusses potential challenges and future directions, including plant-made nanoparticles, phytochemical-mediated modulation of miRNAs, and nanozymes (Vázquez et al.).

Finally, Qian et al., reported an electrospun core–sheath nanofibers, loaded with nanohydroxyapatite (nHA) and simvastatin (SIM), hold potential for bone regeneration applications. These nanofibers, composed of polycaprolactone (PCL), were fabricated using electrospinning technology. Characterization studies confirmed the cylindrical morphology of the nanofibers and the amorphous state of SIM within them. The core–sheath structure enabled sustained release of SIM over 672 h and promoted cell proliferation. This synergistic approach involving materials and nanostructure holds promise for the development of biomedical materials for bone regeneration (Qian et al.).

## Conclusion

In the context of biomedical applications, such as drug delivery, imaging, and tissue engineering, understanding the immunological response to nanomaterials is critical for developing safe and efficacious nanomedicines. Engineered nanoparticles designed for therapeutic purposes must be carefully evaluated to ensure they do not trigger adverse immune reactions or compromise the body’s ability to mount an appropriate immune response against pathogens or cancer cells. Furthermore, the potential immunomodulatory properties of nanomaterials hold promise for applications in immunotherapy and autoimmune disease treatment, as well as vaccine delivery and adjuvant technology. Harnessing the immunomodulatory effects of nanoparticles could lead to novel therapeutic strategies aimed at modulating immune responses for therapeutic benefit. As researchers continue to explore the complex interplay between nanomaterials and the immune system, interdisciplinary collaboration and innovative approaches will be essential for advancing our understanding and harnessing the potential of nanotechnology while minimizing risks to human health and the environment. By integrating immunological principles into the design and evaluation of nanomaterials, we can pave the way for safer and more effective applications of nanotechnology in diverse fields.
